# Global implementation of PrEP for HIV prevention: setting expectations for impact

**DOI:** 10.1002/jia2.25370

**Published:** 2019-08-28

**Authors:** Maria N Pyra, Jessica E Haberer, Nina Hasen, Jason Reed, Nelly R Mugo, Jared M Baeten

**Affiliations:** ^1^ Department of Global Health University of Washington Seattle WA USA; ^2^ Department of Epidemiology University of Washington Seattle WA USA; ^3^ Massachusetts General Hospital Global Health Boston MA USA; ^4^ Department of Medicine Harvard Medical School Boston MA USA; ^5^ PSI Boston MA USA; ^6^ Jhpiego Baltimore MD USA; ^7^ Kenya Medical Research Institute (KEMRI) Nairobi Kenya; ^8^ Department of Medicine University of Washington Seattle WA USA

**Keywords:** HIV prophylaxis, Implementation, Adherence, Uptake, Retention, Prevention

## Abstract

**Introduction:**

Questions remain whether HIV pre‐exposure prophylaxis (PrEP) can be translated into a successful public health intervention, leading to a decrease in population‐level HIV incidence. We use examples from HIV treatment and contraceptives to discuss expectations for PrEP uptake, adherence, and persistence and their combined impact on the epidemic.

**Discussion:**

Targets for PrEP uptake must be based on the local HIV epidemic and will depend on appropriate estimates of the key populations at risk for HIV. However, there is evidence that targets, once established, can successfully be met and that uptake may increase with awareness. Messaging around adherence should include that daily adherence is the goal (except for those MSM for whom event‐driven dosing is a good fit), but perfect adherence should not be a barrier. Ideally, clients persist on PrEP for as long as they are at risk for HIV. While PrEP will be most effective when coverage is focused on high‐risk populations, normalizing rather than stigmatizing PrEP will be highly beneficial.

**Conclusions:**

While many challenges to PrEP implementation exist, we focused on the three key steps of uptake, adherence and persistence as measurable processes that can lead to improved coverage and decreased HIV incidence.

## 1 Introduction

Randomized controlled trials demonstrated that antiretroviral pre‐exposure prophylaxis (PrEP), in pill and vaginal ring forms, is protective against HIV 1, 2, 3, 4. PrEP was first approved for use in 2012 as daily pills and in 2015 global guidelines recommended PrEP as part of effective combination HIV prevention, paving the way for implementation projects in a variety of settings and populations. Nevertheless, because of adherence challenges, including uptake and persistence (i.e. continued PrEP use), in some populations, there have been questions whether PrEP is achieving implementation success and whether it can contribute to reductions in new HIV infections 5. Global use is currently far short of the UNAIDS goal of 3 PrEP million users by 2020, while 1.8 million individuals were infected with HIV in 2017 6 Strong trial results, regulatory approval and clinical guidelines, all of which PrEP has, do not always translate into successful public health interventions.

**Figure 1:**
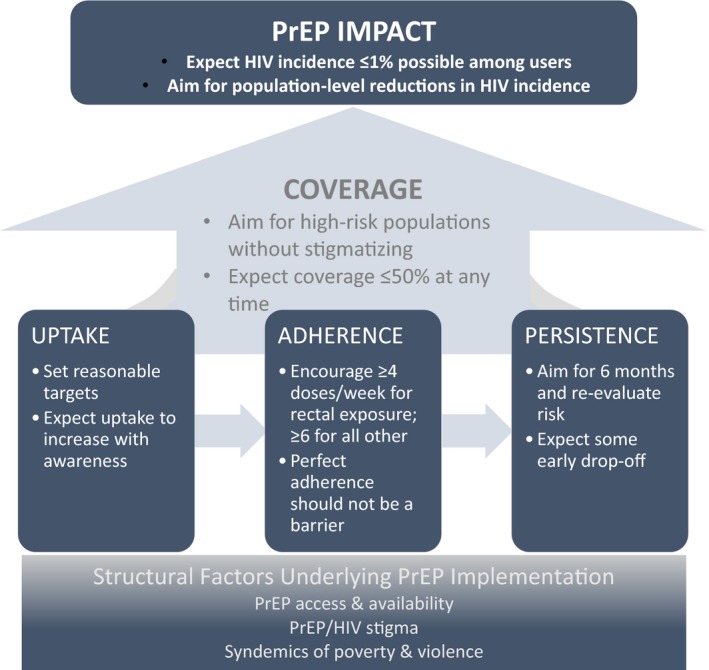
Conceptual framework for PrEP impact.

PrEP is a novel HIV prevention approach – a biomedical intervention for otherwise‐healthy persons requiring at least some continued contact with the healthcare system – with no clear prior model to develop expectations for success. PrEP draws important, but distinct, parallels with ART (another use of antiretrovirals) and contraception (another prevention strategy) that may help inform parameters for success in PrEP implementation. In light of data from PrEP clinical trials and early implementation projects, we consider expectations across key domains – specifically, uptake, adherence and persistence 7 – that can lead to an impact in HIV incidence (Figure 1).

## 2 Discussion

### 2.1 Expectations for uptake

For an individual, PrEP uptake is the first step in achieving protection against HIV. For a population, the number of persons initiating PrEP is the foundation of achieving impact. Of the targeted three million PrEP users, 2018 estimates suggest 300,000 unique PrEP users exist globally, mostly in the United States 8. With antiretroviral therapy (ART), the UNAIDS initiative aims for 90% uptake by 2020. This target uses a fairly straightforward denominator – all persons living with HIV. In contrast, the PrEP denominator is not as easily defined, as HIV risk is hard to assess outside of clinical trials or may not even been known by the participant; for instance, even the CDC's multiplier approach is limited by missing data for risk groups other than MSM 9. PrEP use is not lifelong and depends on risk for HIV, which fluctuates over time. In contraception, a common metric is unmet need, which is determined by the prevalence of several factors, such as pregnancy risk and fertility intention 10, 11 and may be more relevant to developing a PrEP denominator. However, calculating unmet need is a difficult undertaking 12 and there may be more accessible metrics for PrEP, such as the PrEP‐to‐need ratio 13. It is important to note that there is no threshold or goal for PrEP‐to‐need ratios, but it has been used to compare PrEP uptake between key populations 14.

Determining unmet need and uptake targets for PrEP is very much a work in progress. Countries and municipalities have used a variety of approaches to set meaningful uptake targets. In the United States, the CDC estimated the denominator population for PrEP, calculating that 1.1 million individuals meet basic eligibility criteria 9, with only a minority (currently around 220,000) having started PrEP to date 8 and a lively debate exists around improving these criteria 15. Seattle, on a smaller scale, set a target of 50% of high‐risk men who have sex with men (MSM) for PrEP initiation and have reached 36% 16. New South Wales, Australia set a target of 3700 high‐risk users for its PrEP rollout programme, calculated as 9% of high‐risk persons; demand rapidly exceeded expectations and more than 9000 initiated PrEP 17, with an observed 31.5% decrease in new HIV infections in the general MSM population over the following year 18. Uptake was also high in multiple open‐label extension projects 19, 20, 21, 22, 23, suggesting high acceptance among individuals with increased awareness and motivation to use PrEP 16, 24, 25, 26. Most of these results come from MSM, a well‐defined key population in high resource settings; identifying the population at need for women (and other key populations) may face other challenges, including limited resources for research, stigma, gender expectations and the relatively nascent rollout in these populations.

### 2.2 Expectations for adherence

Adherence is key to PrEP's HIV protective effect and was admittedly the Achilles’ heel of some PrEP clinical trials 27. Data from pharmacokinetic studies have helped define potential benchmarks for PrEP use, correlating HIV protection with four or more doses per week for rectal exposure and six or more doses per week for other exposures 19, 28, 29. HIV protection has been nearly complete for those with PrEP detected in their body across a range of settings 19, 23, 30, 31. These data allow for some flexibility regarding event‐driven dosing for MSM, which has demonstrated protection and is recommended in many situations 20, 32, 33.

Setting programmatic benchmarks for adherence is essential for identifying shortfalls for further evaluation and intervention. Daily adherence will be the goal for most individuals 34, 35, but perfection need not be a barrier to potential users 27; event‐driven and seasonal use of PrEP present additional challenges for assessing adherence that require consideration. Given programmatic constraints, adherence should be measured through the most reasonably accessible and preferably objective measure 14, 27, 36. For instance, PrEP adherence (assessed by pharmacy refills) in a large U.S. healthcare system was 92% on average, and no HIV seroconversions were seen 31. In Sydney's rollout programme, adherence was 83% on average as measured by pharmacy refill, with an HIV incidence rate of 0.05 per 100 person‐years (and zero among those actively on PrEP) 37. A range of interventions to improve ART adherence exist, including motivational interviewing, support groups (both real world and online), and text messaging and may be adapted to support PrEP 38, 39.

For ART, 100% adherence is the ideal and an objective measure of adequate adherence is viral suppression. The UNAIDS target is for 90% of those taking ART to be virally suppressed, with current global estimates of 82% 40. For contraception, user‐dependent methods, including condoms and injectables, can prevent upwards of 98% of pregnancies with perfect use, although with typical adherence prevention can be as low as 82% 41. Notably, methods depending less on users, such as implants, tend to have the smallest gap between typical and perfect use 41. As long‐acting injectable PrEP is currently under study, this experience has potential relevance for future PrEP options. For now, contraceptives and ART demonstrate average real‐world adherence resulting in effectiveness on the order of 80% to 90% and this may be a reasonable expectation for PrEP as well.

### 2.3 Expectations for persistence

Once an individual starts PrEP, persistence reflects sustained use; a related concept that prevention programmes may also consider is retention, meaning continued involvement in HIV prevention, such as returning for clinic visits. For ART, indefinite persistence is absolutely essential – individuals benefit only when taking ART. In contrast, for contraception, life‐long, uninterrupted persistence is rarely desired. PrEP falls somewhere between the two, arguably much closer to contraception than to ART.

In theory, the target for PrEP persistence is simple: individuals should take PrEP as long as they are at risk for HIV, a concept known as prevention‐effective adherence 36. However, this concept can be challenging to put in practice for individuals (as mapping risk may be difficult for both PrEP users and providers) and for programmes (since measuring use and risk periods may be difficult to measure and track at scale); financial and systematic barriers may also impede patients from achieving their desired persistence 42, 43, 44. For individuals, risk should be considered in terms of periods or seasons, not days or weeks 45; thus, we suggest an expectation of six months of use, while not a perfect fit for all PrEP users, might be useful for programme evaluation and good habit development 46. A discrete period of planned PrEP use aligns with CDC recommendations that PrEP needs be reevaluated every 12 months 47; a six‐month period is also in line with early implementation projects 30, 32, 48. For instance, the median time on PrEP was 6.3 months for U.S. MSM 49 and in South Africa, 52% of participants had high TFV‐DP concentrations at 30 weeks in the ADAPT study 34. Rates were higher for the dapivirine ring, with 86% using it after six months in one open‐label study 21. However, retention has been lower in some PrEP demonstration projects among some key populations, suggesting an important area for future work 50, 51. Similar to adherence, actually assessing persistence can be challenging, in particular when event‐driven PrEP is used and the time between expected refills is unclear; measures of visit retention may be important to understand persistence as well. Further work understanding event‐driven and seasonal PrEP use is needed.

**Figure 2:**
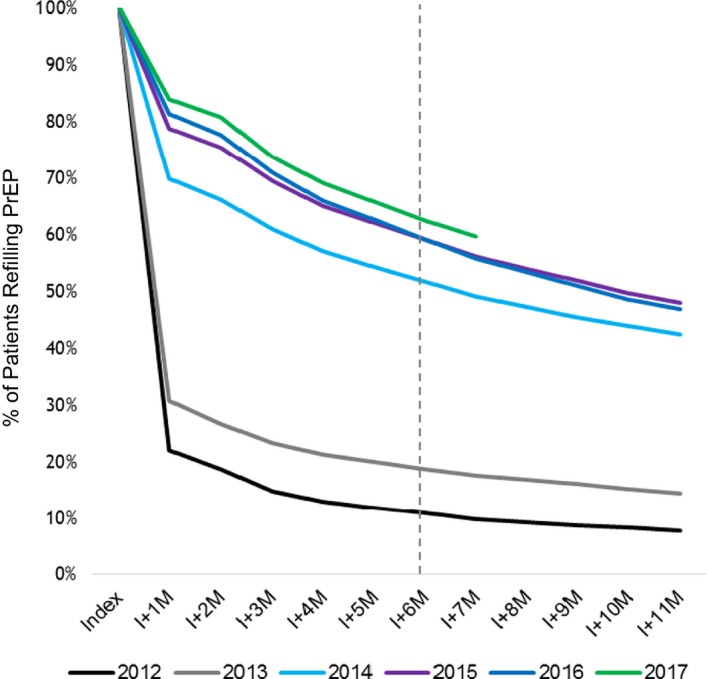
Rapid increase in PrEP persistence among U.S. users, by pharmacy refill Data from 2012 to 2017, a greater proportion of PrEP users were refilling their prescriptions at six months (dotted line). Data courtesy of Gilead Science Inc.

Additionally, there are data to suggest that persistence has been improving since PrEP was first approved. Data from Gilead Science (Figure 2) found only 10% of users remained on PrEP by six months when first introduced, increasing to 50% two years later and >60% by 2017. This change over time suggests that persistence may increase with community awareness of and support for PrEP 52. Whether persistence will show a similar pattern over time in other populations and countries is yet to be seen.

Among individuals living with HIV, the current expectation is lifelong ART persistence, but data suggest only 72% to 89% of those on therapy continue to 12 months 40. Contraceptive continuation rates are even lower: in the United States, 44% of women continued oral contraceptives after six months (by pharmacy refill) dropping to 29% after 12 months; only 40% of women continued with vaginal contraceptive rings by six months 53. Altogether, this data suggests an ambitious aim would be for half or slightly more of PrEP users to continue for six months. However, like contraception, it should not be considered a failure for some individuals to try out a method, discontinue it, try it again, or find another method 54, 55, 56. Importantly, as noted above, PrEP persistence should align with risk for HIV acquisition and some individuals may have shorter, seasonal periods of risk, while still benefitting from PrEP during those periods.

### 2.4 Expectations for coverage leading to impact

The impact of PrEP should be assessed by the overall reduction in HIV incidence not just among users but in the broader population. We define coverage as the composite of uptake and persistence with sufficient adherence on PrEP within the target population, functioning then as the link to impact 57; critically, high uptake without persistence will not lead to impactful coverage. Like prevalence, coverage can be measured at a given point or over a period of time, though this time period should be reported clearly 14, 57. Mathematical models suggest focusing on coverage of high‐risk groups, rather than the general population, is more cost‐effective and in some settings prevents more total infections 58, 59, 60. Even among highest risk groups, complete coverage is not necessarily the goal and is likely not feasible 61, 62, 63, 64. While adherence and drug costs are important questions in modelling, several papers show that even 50% coverage of high‐risk groups can be cost‐effective 61, 64. However, there are benefits to widespread access to PrEP, including normalization of PrEP and better clinical conversations of sexual behaviour 65, and we do not suggest tight restrictions on who can access PrEP.

As noted above, the current UNAIDS goal for ART coverage is 90%, with a recent global estimate of 77% 40. Recent modern contraceptive coverage estimates were 46% among LMIC 66 and 57% globally 67. While the target population for PrEP is smaller than for either contraceptives or ART, it seems reasonable, over time, to expect average PrEP coverage among the at‐risk denominator to be up to 50%, with large variation among countries and populations.

**Figure 3:**
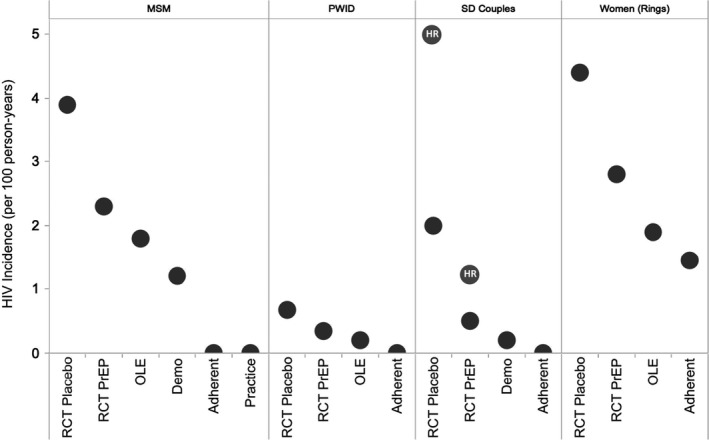
PrEP effectiveness, by study type and population. Compared to placebo populations, HIV incidence (*y*‐axis) decreases among those taking PrEP whether in clinical studies or open‐label settings, across various at‐risk groups: MSM, PWID, sero‐discordant couples, and women. HIV incidence is lowest when PrEP adherence is high. HR =high risk sero‐discordant couples 71; Demo, demonstration project; OLE, open‐label extension; Practice, Real‐world clinical practice; RCT, randomized controlled trial. Data sources: MSM 4, 19, 72, 73, PWID 23, 74, Serodiscordant Couples 3, 30, Vaginal Rings 1, 21, 75.

A notable and unexpected finding of PrEP has been that effectiveness (i.e. HIV protection in open‐label settings) has been higher than efficacy (i.e. protection seen in clinical trials) (Figure 3). A drop in benefit is usually seen with implementation of an intervention as it moves from controlled clinical trials to more open delivery (known as the efficacy‐effectiveness gap) 68, 69. A potential explanation is that once individuals know PrEP works (and are not taking a placebo 70), they are willing to use PrEP and use it well.

Moreover, PrEP benefits extend not only the individual but the overall population, providing something akin to herd protection by reducing secondary infections 76. Rapid population‐level reductions in HIV incidence on the order of 25% to 40% attributable in part to PrEP may already be happening in London, Sydney and San Francisco, based on preliminary, although observational, data 18, 26, 77. With strong uptake, adherence and persistence, data from various open‐label settings (Figure 3) suggest HIV incidence among users can be reduced to around 1% per year or less; among those who are highly adherent, it can approach 0% (with true breakthrough infections carefully investigated to inform future practice).

For ART, the current 90‐90‐90 initiative was derived from models suggesting these benchmarks would lead to an 80% decrease in AIDS‐related mortality and 90% reduction in HIV infections by 2030 78. The impact of contraception on maternal health has been an estimated 44% reduction in maternal deaths globally 79. However, ART and contraception provide secondary benefits as well, such as reducing the number of orphans and improving the economic and educational status of women and their families respectively 80, 81. Secondary benefits for PrEP can be expected as well, such as increased STI testing 82 and early detection of other conditions 83.

## 3 Conclusions

Defining success in PrEP implementation requires appropriate expectations and goals for each step along the path to achieving impact – and the patience to meet these goals. While we have focused on three specific aspects of adherence that are measurable and proximal to population‐level impact, we recognize that many factors – from national and local polices to social influences and the daily challenges facing those most at‐risk for HIV – underlie PrEP implementation and must be addressed (Figure 1). The rollout of PrEP has generally been slow and in some cases challenging, as was true with contraceptives 84 and other HIV prevention methods 85 – although successes in both low‐ and high‐resources settings should not be ignored 37, 86, 87. Through the use of informed, tailored targets around PrEP uptake, adherence, persistence and coverage, as presented in this paper, we can improve PrEP rollout, maximize HIV prevention efforts, and work towards population‐level reductions in HIV incidence.

## Competing interests

JMB, NRM, MNP and JEH have been part of studies with study drug donated by Gilead Sciences.
